# Cancer incidence and mortality trends in young adults in Metropolitan Lima young adults, 1990–2012

**DOI:** 10.3332/ecancer.2020.1025

**Published:** 2020-04-20

**Authors:** Jorge Luna-Abanto, Luis García Ruiz, Jheff Laura-Martinez, Tessy Tairo-Cerron

**Affiliations:** 1 Department of Oncological Surgery, National Institute of Neoplastic Diseases, Lima, Peru; 2 Department of Nuclear Medicine, National Institute of Neoplastic Diseases, Lima, Peru

**Keywords:** young adults, epidemiology, cancer

## Abstract

**Aims:**

The purpose of this research was to calculate and compare standardised incidence and mortality ratios in young adults, based on the data published by the population-based cancer registry of Metropolitan Lima.

**Method:**

A secondary analysis was carried out on the data published by the population-based cancer registry of Metropolitan Lima in its last five volumes. Calculating the standardised incidence ratio, in accordance with the World Health Organization’s standard population, was done using the direct method, and the annual percentage change was calculated using the Joinpoint Regression Program.

**Results:**

From 1990 to 2012, 12,380 new cases of cancer in young adults between the ages of 20 and 49 were reported in Metropolitan Lima. The neoplasms with the highest standardised incidence ratio in the young adult male group were testicular cancer, brain and nervous system cancer, stomach cancer, non-Hodgkin’s lymphoma and bowel cancer. The neoplasms with the highest standardised mortality ratio for this group were stomach cancer, brain and nervous system cancer, non-Hodgkin’s lymphoma, tracheal cancer, bronchial and lung cancer and liver cancer. The neoplasms with the highest standardised incidence ratio in the young adult female group were breast cancer, cervical cancer, thyroid cancer, ovarian cancer and brain and nervous system cancer. The neoplasms with the highest standardised mortality ratio for this group were breast cancer, cervical cancer, stomach cancer, brain and nervous system cancer and non-Hodgkin’s lymphoma.

**Conclusions:**

Young adults represent a highly unique group, characterised by little diagnostic suspicion, distribution and aggressiveness of the neoplasms that occur in them. Assessing and reporting incidence and mortality ratios in this age group can contribute to decision making.

## Introduction

Cancer is a major cause of morbidity and mortality worldwide, regardless of the human development index of populations [1]. According to World Health Organization (WHO) estimates, in 91 out of 172 countries evaluated, cancer is the leading or second leading cause of death before the age of 70 [1]. Data suggests that the global cancer burden has risen to 18.1 million new cases and 9.6 million deaths from cancer [1]. Less than 10% of all newly diagnosed cancer cases in developed countries are known to be adolescents and young adults (YA), this being the most common cause of death in these age groups after homicide, suicide and unintentional injuries [1, 2].

Adolescents and YA have a different distribution of cancer incidence and mortality compared to other age groups [1, 2]. These differences are affected by country, level of economic development, social factors and lifestyles [2, 3]. It is estimated that there are approximately 350,000 to 650,000 new cases of cancer annually for patients aged from 15 to 29, and from 30 to 39, respectively [4]. In selected YA populations in Latin America, the most frequent cancers reported were: thyroid cancer, germ cell tumours, lymphomas, cervical cancer, breast cancer and leukaemia. These findings are similar to other populations worldwide [2–7], and some studies extend the spectrum to colorectal, lung, esophageal and nervous system cancers [2]. On the other hand, a relationship has been reported between mortality from these types of cancer and the human development index, which is inversely proportional to this measurement [8].

The Population-Based Cancer Registry of Metropolitan Lima (RCBPLM), located in the National Institute of Neoplastic Diseases, began its activities in 1968. It currently relies on technical advice from the International Agency for Research on Cancer (IARC) and its inclusion in Volume XI of the Cancer Incidence in Five Continents, as well as GLOBOCAN 2018 reflects the quality of the published data. The latest version published in 2016 contains data from the period 2010–2012 [9].

The patterns of cancer incidence and mortality in YA have been described in depth in other countries, unlike ours, where we do not have studies that address this issue from a population-based perspective. Therefore, this study examined the trends in cancer incidence and mortality in Metropolitan Lima (LM). This information will not only provide a basis for aetiological research, but will also assist in detection and diagnostics, identifying risk factors and setting priorities for cancer control strategies in this economically-active population.

## Methods

### Cancer and patient registry

An ecological study was carried out on trends in cancer incidence and mortality in YA residents in LM. This study was based on a secondary analysis of data published by the RCBPLM in its latest five volumes published for the periods: 1990–1991, 1990–1993, 1994–1997, 2004–2005 and 2010–2012. These volumes are freely accessible and are archived in the National Institute of Neoplastic Diseases library. Digital versions are also available on the website. The RCBPLM systematically and continuously collects data from patients diagnosed with malignant neoplasia during the defined study period, whose normal residence is the city of Lima and the Constitutional Province of Callao. This registry is in association with the IARC and has been included in the publication by the Cancer Incidence in Five Continents [9], for its quality.

During the study period, there were changes in the International Classification of Diseases (ICD), versions 9 and 10, both before and after 1995, respectively. In this population-based cancer registry, tumour histology and behaviour were coded using the International Classification of Diseases for Oncology, Second Edition (ICD-O-2), topography and morphology codes since 2002. Due to the changes in the registry catalogues during this time, all of the cancer diagnosis codes were converted according to the tenth version of the ICD 10.

The patients included in the RCBPLM must present a confirmed diagnosis of some form of cancer, including carcinomas *in situ*, during the period of study [9]. The carrier of the neoplasia must have been a resident of LM for at least 6 months before diagnosis. Incidence data is collected from all of the health institutions in the registry area: public hospitals, the social health insurance system, health services for the armed forces and police, private clinics, pathology and haematology laboratories, and private medical practices. Mortality data was collected from death certificates, registered at the National Registry of Identification and Legal Status of Peru and the Ministry of Health of Peru [9].

The patient population of the age groups under consideration was obtained directly from the population-based registries. For 1990–1991, we took estimations from the National Institute of Statistics and Information (INEI), which are based on previous censuses and published as ‘compendio estadístico 1989–1990’ (statistical compendium). For the period between 1990 and 1993, we used data from the Peruvian national census of 1993. 6,434,323 people from LM were studied, of which 3,145,308 were men and 3,289,015, women. For the period between 1994 and 1997, we used LM’s estimated population based on the 1993 census. We used a polynomial function with Gregory-Newton’s formula to make an estimation as at 31 December 1995. For the period between 2004 and 2005, we used the estimated population as at 31 December 2004. This was taken using a polynomial function with Gregory-Newton’s formula. Finally, for the period of the 2010–2012 registry, we used an estimation from INEI based on the 2007 national census [9]. Population pyramids for young adults are shown in the Supplementary Figures.

### Statistical analysis

The age-adjusted incidence was calculated in the periods reported by the RCBPLM in its five most recent volumes, according to the direct method, taking into account the World Standard Population put forward by the WHO [2–4], by 5-year age groups and expressed per 100,000 people. The total population in LM for the age group between 20- and 49-year old was accounted for on 30th June 1990, 1992, 1995, 2004 and 2011, respectively. This data is available in the volumes published by RCBPLM. We used regression analysis with the Joinpoint programme to identify temporary changes in the incidence and mortality of assessed neoplasias [10]. We calculated the annual percentage change (APC), which shows the variation of incidence in the assessment period. A negative APC indicates a declining trend, while a positive APC indicates an increase in the trend. Trend and frequency graphs were created for the five forms of cancer with the highest incidence and mortality, except skin cancer, for the age group of interest and classified by sex.

### Ethical considerations

Data confidentiality has been maintained according to the guidelines of the Council for International Organizations of Medical Sciences for the analysis of secondary data. It establishes that it is acceptable to use the data for secondary analysis if the intended use comes under the scope of the original informed consent.

## Results

Between 1990 and 2012, 12,380 new cases of cancer in young adults between the ages of 20–49 were reported in LM. Of these, 4,078 were men and 8,302, women. Furthermore, 4,177 deaths by cancer were reported in the same age group: 1,685 men and 2,492 women.

### Incidence in young adult men

**Figure 1.**
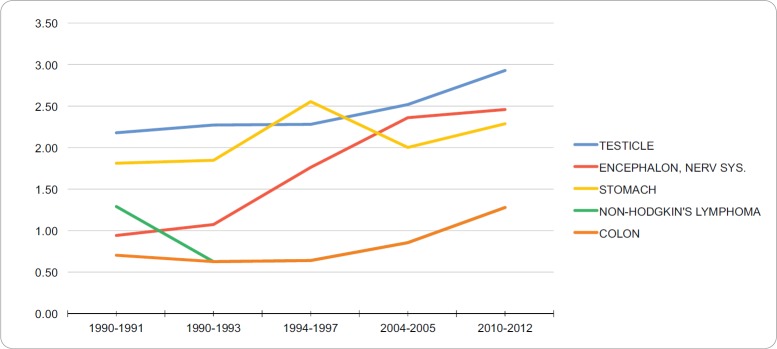
Trend in incidence rates for young adult men (standardized to the WHO world standard population, per 100,000), RCBPLM.

The neoplasias with the highest standardised incidence in the group of young adult men during 2010–2012 were testicular cancer (2.93 per 100,000), cancer of the brain and nervous system (2.46 per 100,000), stomach cancer (2.29 per 100,000, non-Hodgkin’s lymphoma (1.28 per 100,000) and colon cancer (1.28 per 100,000). Table 1 summarises the incidence trend for the standard world population, of the five neoplasias with the highest incidence in this age group between 1990 and 2012. During the years assessed, these neoplasias showed an increase in their incidence. Figure 1 shows the trend of the five neoplasias with the highest incidence in the group of young adult men between 1990–2012. The APC per incident by testicular cancer, brain cancer, stomach cancer, non-Hodgkin Lymphoma and colon cancer in the group of young adult men were 1.25%, 4.56%, 0.63%, 1.34% and 2.97%, respectively. No breakdown points were found (0 Joinpoints) (*p* < 0.05).

### Mortality in young adult men

**Figure 2.**
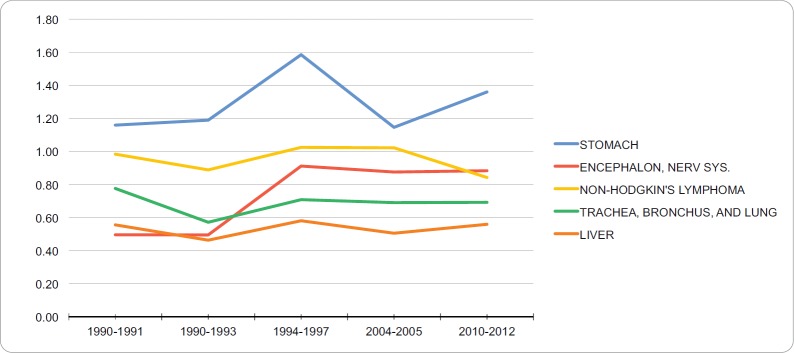
Trend in incidence rates for young adult men (standardized to the WHO world standard population, per 100,000), RCBPLM.

The neoplasias with the highest standardised mortality in the group of young adult men between 2010 and 2012 were stomach cancer (1.36 per 100,000), cancer of the brain and nervous system (0.88 per 100,000), non-Hodgkin’s lymphoma (0.84 per 100,000), trachea, bronchus and lung cancer (0.69 per 100,000) and liver cancer (0.56 per 100,000). Table 2 summarises the mortality patterns for the standard world population of the five neoplasias with highest mortality in this group during 1990–2012. In the years of evaluation, these neoplasias show a trend of slightly increasing. Gastric cancer was the neoplasia with the highest mortality in the studied years. Figure 2 shows the tendency of the five neoplasias with the highest mortality in the group of young adult men between 1990 and 2012. The percentage of annual incident of mortality of young adult men with stomach cancer, brain cancer, non-Hodgkin’s lymphoma, trachea, bronchus and lung cancer and liver cancer were 0.15%, 2.6%, −0.33%, 0.03% and 1.16%, respectively. No breakdown points were found (0 Joinpoints) (*p* < 0.05).

### Incidence in young adult women

**Figure 3.**
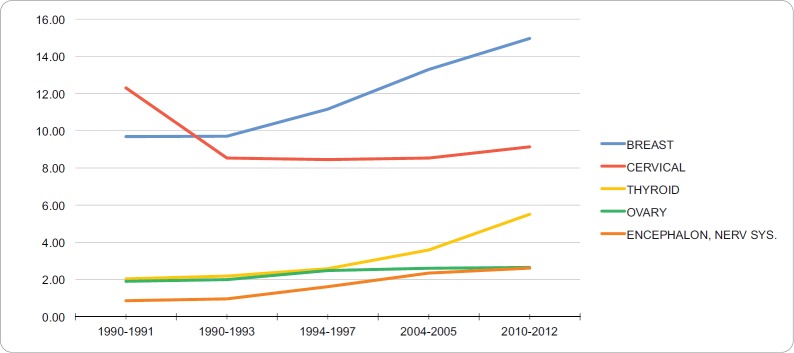
Trend in incidence rates for young adult women (standardized to the WHO world standard population, per 100,000), RCBPLM.

The neoplasias with the highest standardised incidence in the group of young adult women during the period of 2010–2012 were breast cancer (14.97 per 100,000), cervical cancer (9.14 per 100,000), thyroid cancer (5.51 per 100,000), ovarian cancer (2.56 per 100,000) and brain and nervous system cancer (2.61 per 100,000). Table 3 summarises the incidence trends for the standard world population, of the five neoplasias with the highest incidence in this age group during the period of 1990–2012. During these years, there is evidence of a clear upwards trend of the standardised incidence of breast cancer, which started with 9.69 and reached 14.97 affected for each 100,000 young adult women from 2010 to 2012. Cervical cancer had the highest incidence of all neoplasias in the early 1990s, and currently ranks second behind breast cancer, affecting 9.14 young adult women per 100,000. Figure 3 shows the trends for the five neoplasms with the highest incidence in the YA women group from 1990–2012. The APC for the incidence of breast, cervical, thyroid, ovarian, brain and central nervous system cancers in the YA women group were 2.1%, −0.69%, 4.47%, 1.49%, and 5.34%, respectively. No breakdown points were found (0 Joinpoints) (*p* < 0.05).

### Mortality in young adult women

**Figure 4.**
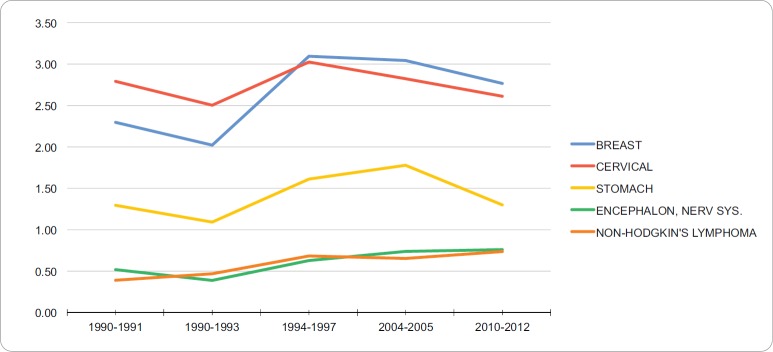
Trend in incidence rates for young adult women (standardized to the WHO world standard population, per 100,000), RCBPLM.

The neoplasms with the highest standardised mortality in the YA women group from 2010–2012 were breast cancer (2.77 per 100,000), cervical cancer (2.61 per 100,000), stomach cancer (1.30 per 100,000), brain and nervous system cancers (0.76 per 100,000) and non-Hodgkin’s lymphoma (0.74 per 100,000). Table 4 summarises the incidence of the five neoplasms with the highest mortality in this group from 1990 to 2012. These neoplasms, mainly breast and cervical cancer, show a decreasing trend during this period. The other neoplasms (gastric and brain cancers, non-Hodgkin’s lymphoma) form a group whose mortality rose in this period. Figure 4 shows trends for the five neoplasms with the highest mortality in the YA women group from 1990 to 2012. The APC in mortality for breast, cervical, stomach, and brain and central nervous system cancers and non-Hodgkin’s lymphoma in YA women were 1.2%, −0.09%, 0.8%, 2.51%, and 2.42%, respectively. No breakdown points were found (0 Joinpoints) (*p* < 0.05).

## Discussion

There are reports on cancer in YA in Peru that address specific neoplasms, such as colon [11], cervical [12] and lung [13] cancers. These studies, however, were limited to evaluating hospital-based cancer registries. This is, therefore, the first study to examine incidence and mortality rates in YA in ML, using a Peruvian population-based registry.

This study documented a total of 12,380 new cases of cancer and 4,177 cancer deaths among YA aged 20–49 in the study period. The specific incidence for the YA group, aged 20–49 years, from 2010 to 2012 was 63 and 132 per 100,000 men and women, respectively. Additionally, the specific mortality in the same period was 21 and 33 deaths per 100,000 men and women, respectively. There are reports that, in this group, new cancer cases among women exceed those in men, but most developed countries do not have the same differences in mortality in [14]. This finding was reported for other series based on population registries, such as those for Shanghai, Australia and Massachusetts [1–5]. This analysis found a higher number of cases among women, up to 1.5 times the number in men. These figures are comparable to earlier data from other series, which found differences in incidence from 1.2 to 1.6 times, in the case of Massachusetts [5]. Some factors that may explain these differences in cancer incidence and mortality are: the immunomodulating effect of sex hormones, the use of exogenous oestrogens and infections. Moreover, the high prevalence of breast, cervical and thyroid cancer in our female population accounts for a large share of cases and merits consideration [4–7]. It is also worth noting that changes in incidence and reduced mortality in YA, such as for cervical cancer, may be related not only to the implementation of public policies (Plan Esperanza [Hope Plan]) but also increased economic resources, access and knowledge of the pathology [15].

### Incidence in young adult men

The most common neoplasm in YA men throughout the study period was testicular cancer. This frequency and higher incidence was also shown in the Australian study by Haggar *et al* [2], who reported a yearly APC of 2.6%. Another study, in the United States, reported that germ cell testicular neoplasm was the most common in this group, calculating an APC of 3.62% for the study period [3]. The known risk factors for testicular cancer are cryptorchidism, testicular atrophy and maternal exposures, but the cause of the sustained increase in this age group remains unknown [2, 16].

The incidence of central nervous system tumours showed the greatest increase during the study period, and an APC of 4.56% was calculated. International reports, conversely, show a nearly constant incidence of this neoplasm [3, 4]. The causes of brain and central nervous system neoplasms are unknown, in addition to the heterogeneous group they represent [16, 17]. Some risk factors have been described, including: exposure to radiation, chemical products, infection; a prior history of cancer, genetic conditions and family histories [5, 18].

Gastric cancer is prevalent in Peru [9], unlike in other regions. A study in the United States analysed the incidence of gastric cancer and found that it rose during the study period. However, sub-classification showed that the incidence of cardial gastric cancer in YA fell in recent years [1]. Our analysis showed that the standardised incidence for YA men was 2.29 per 100,000 from 2010 to 2012. Moreover, the APC was 0.63%, showing very little variation during the study period. It is known that the gastric cancer in YA has a poor prognosis, as the mortality rates discussed later will show [19].

Studies by Bleyer and Keegan [5] found that the incidence of lymphoma in a population is closely related to socio-economic status and exposure to synthetic turf. These studies show an increase in lymphoma incidence in the United States, but did not distinguish between different types [5, 20]. Other studies suggest that lymphoma is the most common neoplasia in YA [7, 21]. In Lima, an increase was found in the incidence of non-Hodgkin’s lymphoma, with an APC of 1.34%. That is comparable to other studies, such as the Chinese (APC: 0.7%) and Australian (APC: 0.5%) studies for the same age group [2, 4].

Colon cancer was the fifth most frequent neoplasm in young adults in Lima, on that note, some studies have estimated an increase in its incidence in Peru [11]. In young adult men from Metropolitan Lima, it was verified that this neoplasm is the one that has the highest annual increase, after neoplasms of the nervous system (APC: 2.97%). Colon cancer is unusual in young adults; however, the highest incidence rates in this population were reported in Chinese males (3.3 per 100,000), while in Latin America an incidence of 2.3 was reported in Bahía Blanca, Argentina [6]. Possible causes of this worldwide increase could be related to low diagnostic suspicion, changes in eating habits, and a higher intake of ultra-processed foods [22, 23].

### Mortality in young adult men

Through our work, it became evident that gastric cancer was the neoplasm with highest mortality in male young adults during the period of 1990–2012. The APC for this neoplasm was 0.15%. Other studies showed similar results, Yin *et al* [24] reported a slight increase of mortality for gastric cancer in this age group. This increase in mortality can be due to the fact that young patients tend to present themselves in advanced stages associated with pathological characteristics of seal ring carcinoma and poorly differentiated carcinoma; these being related to wrong prognostic factors [25].

Brain and nervous system cancer follows in second place, with a calculated APC of 2.6% for the period assessed. Ostrom *et al* [26, 27] also showed that this neoplasm is the second cause of death in males between 20 and 39 years of age in the United States. Another study carried out in Brazil reported a significant increase in mortality from this pathology, with a similar trend than the one in our study [7]. Few reports show a decreased mortality in brain and nervous system cancer. It is possible that this discordant data may be due to advances in the diagnosis and treatment which may not be readily available in some geographical areas [28].

In our study, the third neoplasm with the highest mortality was non-Hodgkin’s lymphoma, with a slight trend towards a decrease, with an APC of −0.33%. However, greater decreases in mortality were reported in other studies [5, 16, 29], which may be related to improvements in treatments with chemotherapy, radiotherapy and bone marrow transplantation [16, 21]. Cancer of the trachea, bronchus and lung took fourth place with an APC of 0.03% this neoplasia displayed a slight increase in mortality. It is expected that, with advances in the treatment, the overall survival of these patients may be improved [29]. Finally, liver cancer was found to take fifth place with a standardised mortality of 0.56 per 100,000 male young adults and a trend towards increase in the last three decades with an APC of 1.16%. Probably, the decrease in this neoplasia which has been highlighted by other studies can be due to an increase in vaccination coverage against the hepatitis virus, which is closely related to this pathology [5, 16].

### Incidence in young adult women

For the time and age group assessed, breast cancer takes first place among cancer incidence in young female adults. This cancer showed an increasing percentage in relationship to the value of 9.69 observed for the 1990–1991 period, and which reached 14,97 new cases for every 100,000 women for the 2010–2012 period. This increase is in line with the current rate of this disease, which according to GLOBOCAN 2018, the standardised incidence for this age range is 34 per 100,000 women [1]. Previous studies in different populations have reported a trend towards an increase in the incidence of breast cancer, due to the increase in screening methods in the last decade; a Chinese study in 2012 determined an APC of 2.9% [4, 30]. Moreover, an Australian study of young adults reported an APC of 0.5% for breast cancer [2].

In second place is cervical cancer, showing a trend towards gradual decrease, with a slight increase in the last period studied; a lower incidence with respect to 13 per 100,000 women currently reported worldwide, this number is comparable with the estimate in other developing countries [1]. Between the decades of the 80’s and the 2000’s the incidence of cervical cancer decreased in developed countries, both in Australia and in the United States [16], where previous studies defined an APC of −2.7% [2]. The incidence of cervical cancer is closely related to a higher incidence of human papillomavirus infection in this age group, and is related to the socio-economic patterns of the populations [30].

Thyroid cancer was the third most frequent pathology, showing a significant and consistent increase during the assessed period. The standardised incidence for female adult young adults was 2.04 between 1990 and 1991, and increased threefold for the period of 2010–2012, where a standardised incidence of 5.51 for each 100,000 women was reported. These results are comparable with those reported in literature, where the incidence of this neoplasm was 11 per every 100,000 female young adults [1, 3]. The percentage of annual variation for the Lima population was 4.47%. This value is comparable to the results in the Australian (APC: 2.1%) and Chinese (APC: 1%) populations [2, 5]. The increased incidence of this neoplasm could be a reflection of better detection, including incidental detection. Female hormonal factors have been identified as a risk factor for thyroid cancer, which could explain the incidence and prevalence of this pathology in the female population. Other risk factors include exposure to radiation, iodine deficiency and family history [16, 31].

Ovarian cancer was the fourth most incident neoplasm. This cancer maintained a stable incidence trend during the assessed period. In our work, an APC of 1.49% was found; other studies have found a lower variation with an APC of 0.3% [1, 2]. Lastly, brain and nervous system cancer showed a progressive and constant increase. A standardised incidence of 0.86 and 2.61 new cases per 100,000 young adult men were reported during the years 1990–1991 and 2010–2012, respectively. By contrast, other studies in different regions in the same age group, reported stable incidence figures in recent decades [2]. The APC is 5.34%, one of the highest reported in the literature, which reports an APC of 1.2% for this neoplasia. The causes of this trend and the risk factors of this neoplasia are unknown [2].

### Mortality in young adult women

Mortality in young adult women is distributed as follows: first, breast cancer, which increased until the period 1994–1997 then decreased to 2.77 deaths per 100,000 women. The APC is 1.2% during the evaluation period, by contrast an Australian study reported a marked decrease in mortality due to breast cancer in this age group (APC: −2.9%) [2]. A recent meta-analysis established an association between obesity and mortality due to breast cancer in pre-menopausal women with a positive oestrogen receptor to the disease, which would be related to dietary habits acquired in the population of young adults [2]. There has been a downward trend in cervical cancer during the period evaluated and a modest decrease in mortality was calculated (APC: −0.09%). A decrease in mortality due to cervical cancer is expected in the coming years as the impact of vaccination becomes apparent, for example, in Australia there has been a large annual decline after it’s widespread use (APC: −6.8%) [2, 30].

Stomach cancer was the third cause of death by cancer in this age group, for the period 2010–2012 the standardised mortality for the age group was 1.3 deaths per 100,000 women, higher figures have been reported in other reports where the mortality for this neoplasia reached 4.9 per 100,000 women [1, 16]. The APC for this period for stomach cancer is 0.8%, other series show better indicators, for example a study of young Chinese adults found an APC of −1.1% [5]. The causes of mortality due to stomach cancer can be explained by an inadequate screening of young adults and diagnosis in advanced stages [3, 5]. The fourth cause is cancers of the brain and the nervous system (APC: 2.51%), the mortality rate of this neoplasia has remained stable during the last three decades [16], other series show a decline in mortality (APC: −4.6%) [4].

Non-Hodgkin’s Lymphoma is the fifth cause of mortality, with 0.39 deaths per 100,000 for the years 1990–1991 and reached 0.74 in the period 2010–2012 (APC: 2.42%). Even though the incidence of this neoplasia is increasing in the world, the mortality rates have declined since the 1980’s (APC: −4.5%) and it is expected that this trend will continue; this better outlook is due to better treatment regimes [7, 21].

### Limitations

This work is not free of limitations, typical of a secondary analysis of collected data. Furthermore, we should consider the parameters for mortality estimation proposed by the WHO in 2003, which indicate that the statistics from recorded mortality can be altered at any stage of their production: data collection and form filling, coding, data processing and subsequent calculations [32]. Mortality rates can also be influenced by migrations, delayed birth registration and change in the disease coding system (ICD), among others. According to WHO reporting, there is a high proportion of under-registration of deaths in Latin America. In this regard, Peru reported 46% under reporting in this publication; however, these results do not reflect the quality of the data collected and corroborated by the RCBPLM team, which are based on direct calculation of rates and not on estimates as proposed by the WHO for Latin America [32].

## Conclusion

This is the first study to investigate the incidence rates or cancer mortality among young adults using a record based on the Peruvian population. Young adults represent a particular group, characterised by the low diagnostic suspicion, distribution and aggressiveness of the neoplasia that presents in them. Changes in the incidence and mortality trend for this age group were found, in relation to lifestyle changes, as well as improved coverage of detection of these neoplasias. We hope that this report can contribute to the design of new strategies that impact the early diagnosis, timely treatment and economic impact of cancer in Peruvian young adults.

## Conflicts of interest

The authors declare that they have no conflicts of interest in the preparation and presentation of this work.

## Funding

None.

## Figures and Tables

**Table 1.** Trend in incidence rates for young adult men (standardised to WHO standard world population, per 100,000), RCBPLM.

Period	1990–1991	1990–1993	1994–1997	2004–2005	2010–2012	APC (%)
Testicle	2.18	2.27	2.28	2.52	2.93	1.25
Brain, Nerv. system	0.94	1.07	2.76	2.36	2.46	4.56
Stomach	1.81	1.85	2.55	2.00	2.29	0.63
Non-Hodgkin’s lymphoma	1.29	0.63	0.64	0.86	1.28	1.34
Colon	0.70	0.63	0.64	0.86	1.28	2.97

**Table 2.** Trend in mortality rates for young adult men (standardised to the WHO standard world population, per 100,000), RCBPLM.

Period	1990–1991	1990–1993	1994–1997	2004–2005	2010–2012	APC (%)
Stomach	1.16	1.19	1.59	1.15	1.36	0.15
Brain, nerv. system	0.50	0.50	0.91	0.88	0.88	2.60
Non-Hodgkin’s lymphoma	0.98	0.89	1.03	1.02	0.84	-0.33
Trachea, bronchus and lung	0.78	0.57	0.71	0.69	0.69	0.03
Liver	0.56	0.46	0.58	0.51	0.56	1.16

**Table 3.** Trend in incidence rates for young adult women (standardised to WHO standard world population, per 100,000), RCBPLM.

Period	1990–1991	1990–1993	1994–1997	2004–2005	2010–2012	APC (%)
Breast	9.69	9.71	11.16	13.29	14.97	2.10
Cervix	12.31	8.53	8.45	8.53	9.14	-0.69
Thyroid	2.04	2.19	2.58	3.59	5.51	4.47
Ovary	1.90	1.99	2.48	2.61	2.65	1.49
Brain, nerv. system	0.86	0.96	1.61	2.34	2.61	5.34

**Table 4.** Trend in mortality rates for young adult women (standardised to WHO standard world population, per 100,000), RCBPLM.

Period	1990–1991	1990–1993	1994–1997	2004–2005	2010–2012	APC (%)
Breast	2.30	2.02	3.09	3.04	2.77	1.20
Cervix	2.79	2.50	3.03	2.82	2.61	-0.09
Stomach	1.29	1.09	1.61	1.78	1.30	0.80
Brain, nerv. system	0.52	0.39	0.63	0.74	0.76	2.51
Non-Hodgkin’s lymphoma	0.39	0.47	0.68	0.65	0.74	2.42
